# T154. RESTING STATE PERFUSION IN THE REWARD SYSTEM LINKED TO DIMENSIONS OF NEGATIVE SYMPTOMS IN SCHIZOPHRENIA

**DOI:** 10.1093/schbul/sby016.430

**Published:** 2018-04-01

**Authors:** Katharina Stegmayer, Andrea Federspiel, Roland Wiest, Sebastian Walther

**Affiliations:** 1 University Hospital of Psychiatry, University of Bern; 2 University Hospital of Psychiatry; 3 University Institute of Diagnostic and Interventional Neuroradiology

## Abstract

**Background:**

Negative symptoms (NS) are central for the symptomatology of schizophrenia associated with poor functional outcome. Two dimensions of NS have consistently been proposed: apathy and diminished expression. Even though distinct pathophysiological mechanism have been hypothesised resting state perfusion and dimensions of NS have not been studied. Here, we therefore focused on dimensions of NS and the link to whole brain resting state perfusion in schizophrenia patients.

**Methods:**

We included 45 schizophrenia spectrum patients and 44 age- and gender-matched healthy controls. We assessed NS with the Scale for the Assessment of Negative Symptoms (SANS) and imaging on a 3T MRI scanner. Apathy was currently present in 31 patients and diminished expression in 27 patients. Patients did not differ in antipsychotic medication or positive symptoms. We compared whole-brain perfusion over all, and between the groups using 1-way ANCOVAs (F and T tests). A uniform threshold of p < 0.5 (FWE-corr) was applied.

**Results:**

Diminished expression was most prominently associated with perfusion within the right orbital cortex, insula, ventral striatum and head of caudate nucleus, while apathy was associated with perfusion bilateral within the SMA, the insula and the thalamus.

**Discussion:**

Dimensions of NS at rest were associated with altered resting state perfusion, in particular in brain areas relevant for reward processing. Distinguishable associations of rCBF with NS dimensions point to distinct underlying pathophysiology.

